# Evolutionary implications of interspecific variation in a maternal effect: a meta-analysis of yolk testosterone response to competition

**DOI:** 10.1098/rsos.160499

**Published:** 2016-11-30

**Authors:** Alexandra B. Bentz, Daniel J. Becker, Kristen J. Navara

**Affiliations:** 1 Poultry Science Department, University of Georgia, 203 Poultry Science Building, Athens, GA 30602, USA; 2 Odum School of Ecology, University of Georgia, 140 E. Green Street, Athens, GA 30602, USA

**Keywords:** avian, life-history traits, coloniality, nest type, aggression

## Abstract

Competition between conspecifics during the breeding season can result in behavioural and physiological programming of offspring via maternal effects. For birds, in which maternal effects are best studied, it has been claimed that exposure to increased competition causes greater deposition of testosterone into egg yolks, which creates faster growing, more aggressive offspring; such traits are thought to be beneficial for high-competition environments. Nevertheless, not all species show a positive relationship between competitive interactions and yolk testosterone, and an explanation for this interspecific variation is lacking. We here test if the magnitude and direction of maternal testosterone allocated to eggs in response to competition can be explained by life-history traits while accounting for phylogenetic relationships. We performed a meta-analysis relating effect size of yolk testosterone response to competition with species coloniality, nest type, parental effort and mating type. We found that effect size was moderated by coloniality and nest type; colonial species and those with open nests allocate less testosterone to eggs when in more competitive environments. Applying a life-history perspective helps contextualize studies showing little or negative responses of yolk testosterone to competition and improves our understanding of how variation in this maternal effect may have evolved.

## 1. Introduction

Competition is a selective force continuously shaping individual phenotypes and populations [1–3]. Aggression—or the threat of aggression—is usually needed to be successful in competitive interactions [1], and while aggression can be beneficial if it secures resources, there is invariably a cost (e.g. time and energy or increased risk of injury and pathogen transmission [4,5]). The strength of competitive aggression as a selection pressure can depend on variation in the social environment (e.g. breeding density [6]), and this environmental heterogeneity can potentially elicit adaptations that span generations via maternal hormones [7]. Hormone-mediated maternal effects are a non-genetic source of phenotypic variation in offspring exposed to maternal hormones during periods of high developmental plasticity, such as embryonic development [8,9]. Maternal effects are common across taxa (e.g. mammals [10] and fish [11]), but are best studied in birds because maternal hormones are deposited in externally developing eggs [7]. The majority of studies examining intraspecific variation in avian maternal hormone allocation focus on the effects of competitive social interactions [7]. Most of these studies find a positive relationship between testosterone allocated to egg yolks and transient increases in competition, such as increased breeding density [12–18] or conspecific aggression [19–23]. Exposure to increased yolk testosterone typically increases nestling development (e.g. [24–28], but see [7]) and aggressive behaviours throughout life [29–31]. This has led researchers to postulate that yolk testosterone allocation is an adaptation to competitive environments [32].

Maternal effects may be an adaptive means by which females change the phenotype of offspring in preparation for the current environment [7,32], specifically by increasing juvenile survival in the maternal environment [33] and/or success as adults if they remain in or select a similar habitat to that of the maternal environment [34]. The phenotypic changes in offspring associated with yolk testosterone, like increased aggression, seem beneficial in high-competition environments; however, not all studies show a positive relationship between competitive environment and yolk testosterone [35–44]. Authors of one study showing a negative relationship between breeding density and yolk testosterone suggested these results were an artefact of more aggressive birds defending larger territories and creating lower densities; alternatively, their measure of density could have been confounded with vegetation height, making it difficult to interpret the results [40]. Yet, other studies have also failed to support a positive relationship between yolk testosterone and competition, and authors have offered varying reasons for these findings, including high incidence of extra-pair mating [36] and coloniality [42]. Life-history traits could plausibly cause innate differences in competitive environments that shape how a species responds to transient changes in competition, such as seasonal variation in breeding density. Furthermore, to fully determine if this maternal effect is an adaptation to transient changes in competitive environments, a comparative phylogenetic approach is necessary to show how maternal effects have evolved with changes in innate levels of competition across species [45].

Interspecific variation in yolk testosterone may exist due to evolutionary constraints arising from life-history traits; however, few studies have explored interspecific variation in yolk testosterone. Those that have taken comparative approaches showed that average yolk testosterone concentrations per bird species are related to nestling development [46,47] and aspects of songs [48]. Gil *et al*. [49] performed one of the most comprehensive comparative studies and showed that colonial bird species allocate more yolk androstenedione, the biologically inactive precursor to testosterone, but not testosterone, than solitary species. Though this body of work has examined species differences in average yolk testosterone concentrations, investigation of the evolution of a purportedly adaptive maternal effect requires consideration of environmental context; therefore, change in maternal-derived yolk testosterone values rather than average concentrations are required. We present here the first comparative approach of a maternal effect by asking if effect size (i.e. maternal response to competitive environments) varies across species based on life-history traits that evolved with potentially different innate levels of competition. To test this, we performed a meta-analysis with yolk testosterone allocation effect size in response to competition in 25 intraspecific avian studies. We tested whether and how different life-history traits account for interspecific variation in yolk testosterone allocation. If intraspecific variation in yolk testosterone allocation has evolved to be adaptive for competitive environments, we would expect to find this maternal effect related to life-history traits that potentially influence interspecific variation in competition.

## 2. Material and methods

### 2.1. Literature search

We performed systematic searches in Web of Science, Google Scholar, JSTOR and PubMed. We used the same string of search terms in all databases: (avian OR bird) AND (‘yolk testosterone’ OR ‘egg testosterone’ OR ‘yolk androgen’ OR ‘egg androgen’) AND (aggression OR ‘simulated territorial intrusion’ OR ‘breeding density’ OR ‘social environment’ OR ‘colony size’ OR ‘maternal environment’). We restricted searches from the date of the search, 5 September 2016, back to 1993, when the first discovery of maternal yolk testosterone allocation was made by Schwabl [50]. We only included observational or experimental studies comparing yolk testosterone allocation across variation in environments eliciting competitive behaviours in breeding females following a systematic exclusion process ([51]; see electronic supplementary material, figure S1). Studies that only presented data for androstenedione, the biologically inactive precursor to testosterone (e.g. [52]), or examined yolk testosterone relationships with social hierarchy, which is not an environmental manipulation (e.g. [53,54]), were excluded.

We ultimately included 22 articles in our meta-analysis that recorded measures of yolk testosterone concentration response to competitive aggression. Two articles (i.e. [16,40]) performed studies in two different years and analysed these data separately, so we included both years. We also augmented our sample size with an unpublished dataset (A.B. Bentz, V.A. Andreasen & K.J. Navara 2015, unpublished data) involving the experimental manipulation of zebra finch (*Taeniopygia guttata*) pairs' competitive environment using conspecific intrusions (*n* = 16 pairs) to bring the final sample size to 25 records. Our criteria for inclusion in the meta-analysis resulted in a small sample size, and conclusions regarding yolk testosterone allocation response to competition should be regarded cautiously. However, given the low number of available studies of this phenomenon, we hope our meta-analysis will prompt future avian work within a new life-history and phylogenetic framework.

### 2.2. Effect size calculation

We reported effect sizes as the correlation-based *r* between our factor (i.e. competitive aggression) and response (i.e. yolk testosterone allocation). In most cases, *r* was not reported; therefore, we followed Rosethal & DiMatteo [55] to convert test statistics (e.g. *F*, *t* or *χ*^2^) into *r* (but see [56]). We primarily calculated *r* using *F* and the error d.f., when the numerator d.f. was 1:

2.1 $$r=\sqrt{\frac{F}{F+\text{d.f}._{\mathrm{error}}}.}$$

If there was more than 1 d.f. in the numerator or a random effect was included, we converted the reported *p*-value to a standard normal deviate *Z*-score and used the sample size to obtain *r*:

2.2 $$r=\frac{Z}{\sqrt{N}}.$$

For our analyses, we assigned a negative value to effect sizes for which the independent variable was negatively related to yolk testosterone. However, we were unable to determine directionality of effect for four studies with non-significant results (e.g. only an *F* statistic and *p-*value were reported); therefore, we performed our primary analyses with both negative and positive values assigned for these records, though only results from the case of positive values are depicted. Directional *r* effect sizes were converted to Fisher's *Z* to stabilize variance. We used the R package *metafor* for *r*-to-*Z* effect size conversions [57].

### 2.3. Selection and coding of moderator variables

We selected moderators based on previous interspecific comparisons [46–49] and life-history traits that have the potential to influence the adaptive context for the direction or strength of the relationship between level of competition and yolk testosterone response (i.e. effect size). Coloniality (i.e. solitary, semi-colonial or colonial) can influence the frequency of intraspecific competition [58,59], nest type (i.e. open- versus closed-nesters) can influence nest competition and predation risk [60–63], parental investment (i.e. a multiple correspondence analysis of clutch size, altricial versus precocial nestlings and time to fledge; [64]) needed in biparental species (none of the species in this study exhibit male- or female-only care) can influence intra-sexual mate competition [65,66], and mating type (i.e. polygamous/cooperative breeders versus monogamous) can affect intra-sexual mate competition [67] (see electronic supplementary material for references and details on moderator coding, electronic supplementary material, table S1). Because monogamous species are not always truly monogamous [68], we also calculated degree of monogamy when data were available (*n* = 10 species) using the weighted average of percentage of nests that have extra-pair young across a minimum of two populations (see electronic supplementary material, table S1). We also considered experiment type, as studies differed in how they measured and/or elicited competitive aggression (correlative, no manipulation; indirect manipulation of aggressive interactions by changing environment; or direct manipulation of aggressive interactions with simulated territorial intrusions).

### 2.4. Model selection and hypothesis testing

We used random effect models (REM) to estimate the average true effect size and heterogeneity among effect sizes, and univariate mixed-effects models (MEM) to test how each life-history trait moderates the relationship between competition and yolk testosterone response [69]. Separate MEMs with observation, study and species included as random effects (see ‘Controlling for phylogenetic signal’ below for details on the species component) were built to account for unit-level heterogeneity, study pseudoreplication and phylogeny while explaining variation in effect size according to moderator variables [70,71], using the *metafor* package in R [57]. To assess relative support for each hypothesis, we first fit models using maximum likelihood and calculated AICc to correct for the small sample size [72]. We next refit these models using restricted maximum likelihood to obtain unbiased estimates of variance components and tested if each moderator explained significant heterogeneity with Cochran's *Q*. We also assessed the relative contribution of true heterogeneity to the total variance in effect size through the *I*^2^ statistic [71,73]. To quantify the variation in effect size explained per moderator, we calculated the proportional reduction in the summed variance components from each MEM compared with the summed variance components of the REM, equivalent to a pseudo-*R^2^* value [74,75]. For models within 2 ΔAICc of the best-fit model [72], we performed *post hoc* comparisons adjusting for the potentially inflated false-discovery rate using the Benjamini and Hochberg correction [76,77]. In a secondary analysis, we built an MEM for per cent extra-pair copulations and performed the same set of analyses, as data for this moderator was available for a subset of records (*n* = 16 records comprising 10 species). We tested residuals for normality and examined profile likelihood plots of variance components to ensure parameter estimates were identifiable.

Meta-analysis models include weighting by sampling variances, which results in more precise estimates of coefficients and increases power, even when sample size is small [78,79]. However, due to our small sample size, we limited the number of models to under one-third of our *n* [72].

### 2.5. Controlling for phylogenetic signal

**Figure 1.**
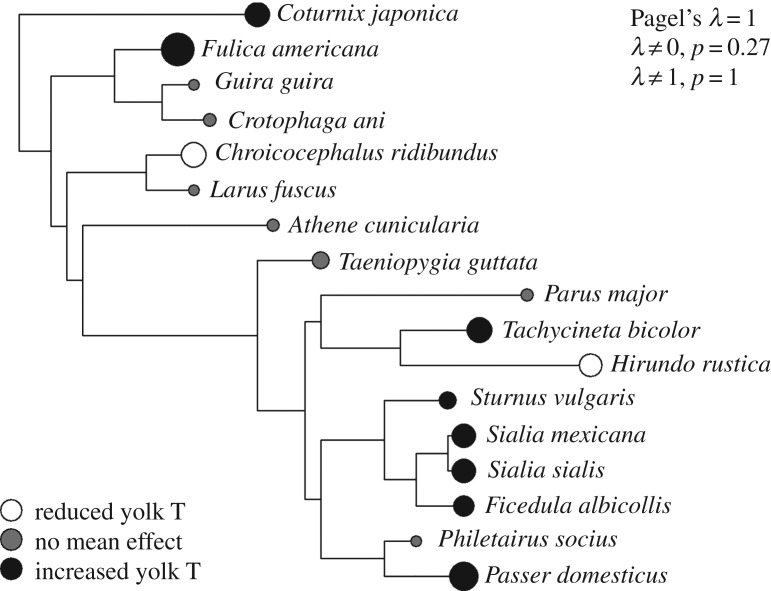
Phylogenetic visualization of mean yolk testosterone response per species included in the main analyses. The displayed tree was pruned from the complete tree of life [82], with each point representing the weighted average effect size per species. Circle size represents the magnitude of the relationship between yolk testosterone response and competitive environment, and the directionality of this relationship is given in the key and is based on the significance of findings from their respective studies. Results from likelihood ratio tests for phylogenetic signal (Pagel's λ) are provided in the legend.

Closely related species may have similar yolk testosterone responses to competitive environments [80]. We first obtained a phylogeny using the R package *ape* to prune the complete tree of life to our 17 species [81,82]. The mean effect size per species was calculated by weighting each observation by the corresponding sample size. We determined phylogenetic signal using maximum likelihood to estimate Pagel's λ [83] and compared the fit of this model against those in which λ was set to 0 (phylogenetically uncorrelated) and 1 (phylogenetically dependent) using likelihood ratio tests in the R package *geiger* [80,84]. Within our tree, we found evidence for phylogenetic dependence in effect size (figure 1). The maximum-likelihood estimate of λ was 1, and likelihood ratio tests suggested this estimate did not differ from the Brownian motion model of trait evolution (*χ*^2^ < 0.001, d.f. = 1, *p* = 1.00). Yet we could also not reject that λ departed from a model with no phylogenetic signal (*χ*^2^ = 1.21, d.f. = 1, *p* = 0.27). To incorporate all 25 records while accounting for phylogenetic non-independence in the above MEM analyses, the covariance structure of the species random effect was specified by the correlation matrix of our phylogeny, equivalent to a phylogenetic meta-analysis [57,70,71].

### 2.6. Publication bias

We also tested for publication bias, the preferential publication of significant over non-significant results or those with a small effect size [85]. We used a funnel plot to visualize potential bias, where a symmetrical funnel suggests little bias [86]. We assessed symmetry with regressions to test associations between effect sizes and sampling variances [87]. We then used the trim-and-fill method to estimate the number of observations missing owing to publication bias and tested if addition of these points influenced REM estimates [57,88].

## 3. Results

### 3.1. Effect of moderators

**Figure 2.**
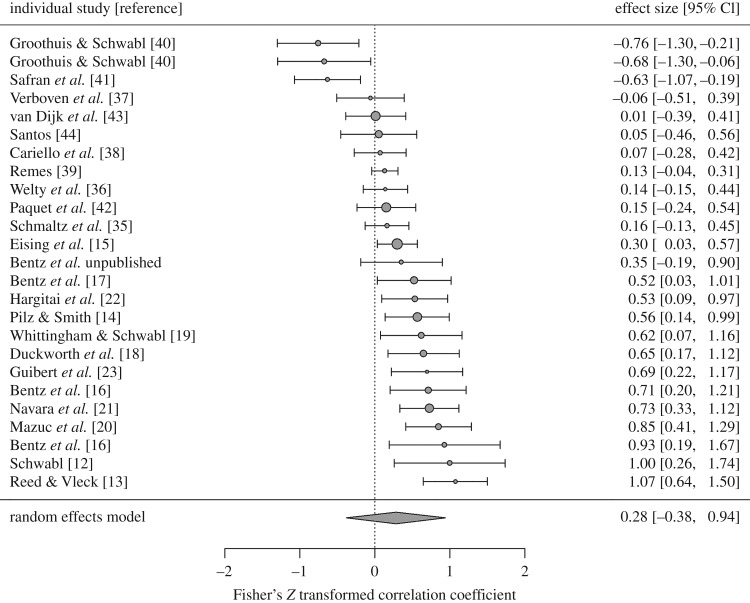
Distribution of effect sizes for relationships between competitive environment and yolk testosterone response (Fisher's *Z* ± 95% CIs). Circle size is scaled inversely proportional to the sampling variance, and points to the left of the dashed line indicate cases where competitive environment was associated with decreased yolk testosterone. The diamond displays the estimated true effect from the multilevel REM fit with restricted maximum likelihood.

We found significant heterogeneity in avian yolk testosterone responses to competitive environments (*I^2^* = 0.92, *Q* = 95, d.f. = 24, *p* < 0.0001; figure 2), with 52% of studies finding that females significantly increase yolk testosterone in more competitive environments (*n* = 13), 12% of studies finding significant decreases (*n* = 3) and 36% finding no significant effect (*n* = 9). The REM showed an average positive but non-significant effect size (*μ* = 0.28, *z* = 0.83, *p* = 0.41), probably owing to the large variance attributed to phylogeny $\left(\sigma_{1}^{2}=0.44\right)$.

Model comparison by AICc lent the most support for species coloniality and nest type as the strongest predictors of yolk testosterone response to competition, as the cumulative Akaike weight of these models exceeded 90% (table 1*a*). Coloniality explained 55% of the variation in effect size compared with the base REM (*Q*_M_ = 14.85, d.f. = 2, *p* < 0.001), and nest type explained 29% of effect size variation (*Q*_M_ = 6.70, d.f. = 1, *p* = 0.01; table 1*a*). Only these two moderators performed better than the REM when compared with other species- and study-specific moderators. The ranking of models by AICc was identical when we assigned negative signs to the four effect sizes where directionality could not be determined and lent even more support to coloniality as the best predictor of effect size. Thus, the presented results using positive values for those four effect sizes could be interpreted as conservative. Our secondary analysis for degree of monogamy (% EPC) found that this trait was not associated with effect size (table 1*b*). All model residuals were normally distributed (*W* ≥ 0.92, *p* ≥ 0.06) with the exception of degree of monogamy (*W* = 0.81, *p* < 0.01), and profile likelihood plots of the variance components indicated strong parameter identifiability. Variance for the species-level random effect $\left(\sigma_{1}^{2}\right)$ ranged from 0.20 to 0.49. By contrast, variance components for the study- and observation-level random effects ($\sigma_{2}^{2}$ and $\sigma_{3}^{2}$, respectively) were consistently zero. Accordingly, a large proportion of the unaccounted variance in effect size was due to residual heterogeneity from phylogeny ($I_{\mathrm{species}}^{2}$ ranged from 0.82 to 0.92).

**Table 1.** Univariate rankings of mixed-effects models (MEMs) predicting effect size for the relationship between competitive environment and yolk testosterone response for the (*a*) full and (*b*) reduced dataset. Competing models are ranked by AICc along with the number of model coefficients (*k*); variance components for the species $\left(\sigma_{1}^{2}\right)$, study $\left(\sigma_{2}^{2}\right)$ and observation random effect $\left(\sigma_{3}^{2}\right)$; *I*^2^ statistic, tests of moderator significance (Cochran's *Q* and *p* value); Akaike weights (*w_i_*) and the pseudo-*R^2^* statistic for each MEM.

mixed-effects models	*k*	$\sigma_{1}^{2}$	$\sigma_{2}^{2}$	$\sigma_{3}^{2}$	*I*^2^	*Q*_M_	d.f.	*p-*value	ΔAICc	*w_i_*	*R*^2^
(*a*) full dataset (*n* = 25)											
effect size ∼ coloniality	3	0.20	0.00	0.00	0.82	14.85	2	<0.001	0.00	0.78	0.55
effect size ∼ nest type	2	0.32	0.00	0.00	0.88	6.70	1	0.01	2.98	0.17	0.29
effect size ∼ intercept	1	0.45	0.00	0.00	0.92	0.69	1	0.41	6.41	0.03	0.00
effect size ∼ mating type	2	0.48	0.00	0.00	0.92	0.14	1	0.71	9.41	0.01	0.00
effect size ∼ parental effort	2	0.49	0.00	0.00	0.92	0.00	1	0.96	9.57	0.01	0.00
effect size ∼ experiment type	3	0.35	0.00	0.00	0.89	2.49	2	0.29	10.53	<0.01	0.22
	(*b*) reduced dataset (*n* = 16) for per cent extra-pair copulations (% EPC)										
effect size ∼ logit (% EPC)	2	0.48	0.00	0.00	0.92	0.53	1	0.47	—	—	0.00

**Figure 3.**
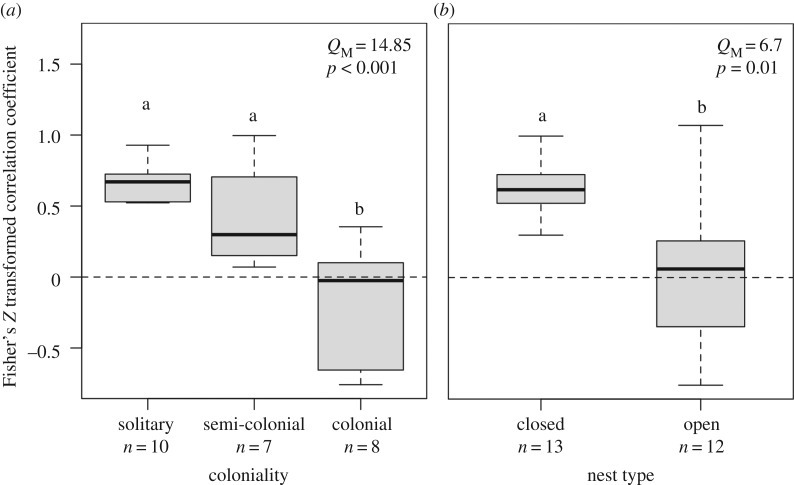
Distribution of effect sizes according to (*a*) coloniality and (*b*) nest type. Boxplots show the median and first and third quartile of the effect sizes, and whiskers indicate the range of non-outlier values. The dashed horizontal line represents no yolk testosterone response to competitive environment. Letters indicate significant differences between groups after adjusting for the potentially inflated false-discovery rate using the Benjamini and Hochberg correction. Results of the omnibus tests match those from the full dataset and analyses in table 1. Sample size (*n*) indicates the number of records per category.

Our best-supported MEMs independently included coloniality and nest type. Adjusting for multiple comparisons, we found that colonial species had lower yolk testosterone responses than both solitary and semi-colonial species (all *z* < −2.6, *p* < 0.02; figure 3*a*). We likewise found a strong overall effect of nest type on yolk testosterone response to competition, with lower effect sizes for species with open nests than closed nests (*z* = –2.59, *p* = 0.01; figure 3*b*).

### 3.2. Publication bias

We found little evidence of publication bias in the study of avian yolk testosterone response to competition (see electronic supplementary material, figure S2). We did not detect an association between effect size and standard error (*z* = 0.80, *p* = 0.42), and trim-and-fill analyses using the R0 estimator did not detect any missing effect sizes. These results suggest a lack of the ‘file drawer’ effect [85]; research in this field was earnestly published regardless of effect size or statistical significance.

## 4. Discussion

Interspecific variation in female testosterone allocation to eggs as a response to competitive environments was strongly predicted by two life-history traits, coloniality and nest type. It should be noted that no colonial breeders also had closed nest types in our dataset; therefore, despite analysing these separately, it may be difficult to say which of the two life-history traits is truly the stronger driver of effect size. However, the *R*^2^ for coloniality was far higher than that of nest type, supporting the argument that coloniality is the stronger of the two. The majority of colonial species (three of five species) in our limited dataset also have altricial development types, meaning only two colonial species were also precocial. Given the current sample size, we cannot test for an interaction between coloniality and development type; however, the adaptive value of yolk testosterone could vary as precocial nestlings face direct competition from peers in higher densities (unlike altricial nestlings). Additional data for precocial species are, therefore, urgently needed. Nevertheless, we more broadly found no support for development type as a determinant of effect size, suggesting that the observed relationship between coloniality and yolk testosterone allocation in response to competition is driven more directly by features of colonial life history. That experiment type was not a strong predictor of this maternal effect also suggests correlative studies were able to obtain an adequate range of competition and that those studies directly inducing competition did so within the natural range. Further, strong phylogenetic signal in effect size provides support for selection on yolk hormone allocation strategy as an adaptation to species-specific competition.

### 4.1. Coloniality

The life-history trait that received the most support in our analysis was coloniality. Colonial species deposited less yolk testosterone in response to competition than either solitary or semi-colonial species. Solitary and semi-colonial species did not differ in yolk testosterone response, though there was a trend for semi-colonial species to deposit less yolk testosterone. While we did not include Gil *et al*. [52] in the meta-analysis because they reported the relationship between colony size and yolk androstenedione, not testosterone, it is interesting to note that they did not find a significant relationship between yolk androstenedione and colony size in the highly colonial barn swallow (*Hirundo rustica*).

The costs and benefits associated with colonial breeding create trade-offs and selective pressures unique from those under which solitary species have evolved. Most notably, colonial species have a higher frequency of conspecific interactions [59,89,90]. Aggression between conspecifics can lead to physical harm and increase reproductive costs, such as egg loss [91,92]. Thus in colonial species, for which opportunities for competitive interactions are much more frequent, the potential adaptive value of reducing the severity of these aggressive interactions should be high. For example, current fitness is higher in colonial birds that allopreen neighbours (an altruistic behaviour) at a higher rate and have fewer fights than those that do not express this behaviour [93]. Indeed, there are several examples of adaptations in social species that decrease severity of aggression compared with solitary species, such as increased attack latencies [94], increased number of appeasement signals [89] and altered neural non-apeptide responses to aggression [95]. The transition to coloniality could have created selection for a decrease in amplitude of aggressive actions, which would be facilitated by a lower yolk testosterone response creating offspring with less aggressive phenotypes. Within many colonial species, increases in breeding density are accompanied by more frequent interactions with neighbours but fewer interactions escalate to high levels of aggression [89,96]. By contrast, aggression is less frequent for solitary species, and the burden of the cost is dispersed over time so the adaptive value of being aggressive is probably much higher [97]. This would favour increased yolk testosterone in response to competitive environments to increase offspring aggressive phenotype.

Another explanation for attenuated yolk testosterone responses in colonial species could be selective pressures imposed by greater parasite risk. Colonial species live in close proximity to one another and have a higher risk of pathogen infection and ectoparasitism, which has direct fitness effects on mortality and more subtle effects on fecundity [98,99]. Hence, there could be strong selection to allocate less yolk testosterone when colony size is large and risk of parasitism is high, because yolk testosterone may decrease immunity and increase host susceptibility [7,26,100].

### 4.2. Nest type

Species with open nest types deposited less yolk testosterone in response to competition than those nesting in closed nests, like cavities. One explanation for this could be that birds with open nests suffer greater nest predation than those nesting in cavities [60,62]. Predation tends to select against bold, aggressive individuals and forces individuals to re-allocate time and energy away from competition toward predator avoidance [63,101]. Females of species exposed to greater levels of nest predation may have adapted a more subtle response to competitive challenges, as shown here. An experimental study found females exposed to frequent predation during egg laying deposit less testosterone into their eggs [102]. Yolk testosterone generally increases growth and begging rates, so by not allocating more yolk testosterone despite having a more competitive environment, females could be using an adaptive strategy to reduce revealing traits (i.e. larger, louder offspring) and thus avoid detection by predators [103]. However, a meta-analysis of Passeriformes suggests that yolk testosterone allocation may increase with predation rate, possibly to accelerate development and reduce exposure to predation in the nest [47]. Thus, an alternative explanation for our findings is that cavity-nesting birds may compete more for limited nest sites compared with open-nesting birds [61] and, therefore, have adapted strategies to increase aggressive phenotypes when nest site competition is high [97], such as increasing yolk testosterone with breeding density.

### 4.3. Potential mechanisms

Researchers have previously postulated that female plasma and yolk hormone responses to aggression were positively correlated [104,105], leading to the assumption that competition should increase yolk testosterone. Yet not all studies show a positive relationship between plasma and yolk testosterone in response to aggression [21,106]. Furthermore, there is a trend for both solitary and colonial species to increase plasma testosterone with conspecific aggression [89,107], yet we show here that these groups differ in yolk testosterone allocation. Colonial females also tend to have higher circulating testosterone than solitary females [108], but their egg yolks do not have higher average concentrations of testosterone [49]. The mechanisms regulating yolk and plasma testosterone could, therefore, be independent [109,110]. For example, differences in expression of follicular steroidogenic enzymes, such as aromatase, explain substantial variation in yolk testosterone but not in plasma testosterone [111]. Expression of steroidogenic enzymes can change rapidly in response to environmental factors [111,112] and may be a point of selection for yolk testosterone response to competition. By contrast, plasma testosterone may originate from multiple sources, such as the gonad and adrenal glands, and new research asserts that steroids can be produced rapidly and locally in the brain in response to aggression [113,114], which would operate independent of follicular production. Other factors could also influence yolk allocation independent of plasma, such as metabolic conjugation [115] or enzymatic barriers or membrane transporters to alter steroid transfer from follicles to the yolk [116]. There is also evidence that natural selection can shape yolk and plasma hormone allocation separately. Yolk testosterone concentrations have a heritable component (e.g. [117–119]), and yolk and plasma testosterone respond to artificial selection differently [110].

If yolk and plasma testosterone are moderated independently and have a heritable component, then selection on mechanisms moderating plasma testosterone may have occurred to benefit females as they respond to aggressive interactions; while selection on mechanisms moderating yolk testosterone may have occurred more in response to offspring success. Few studies have explicitly tested fitness consequences (survival or reproductive success) of prenatal exposure to yolk testosterone. There is mixed support for yolk testosterone's effect on early survival in both altricial and precocial offspring (reviewed in [7]); however, few of these studies incorporated an environmental context or selection pressure known to influence yolk testosterone allocation. One such study found that yolk testosterone increases survival of offspring in poor conditions ([33], but see [120]). Further, because the effects of yolk testosterone on aggression last into adulthood [29–31], selection can occur on adult offspring if they remain in or select a similar habitat to that of the maternal competitive environment. There is some evidence habitat choice has a heritable component [34]. Regardless, many studies find controversial effects of yolk testosterone on adult survival [121,122] and reproductive success [123,124], yet these studies do not manipulate the environmental context. To further elucidate the life stage in which selection on yolk testosterone allocation may occur, more studies should combine egg hormone injections with environmental manipulations. Studies in other taxa, such as invertebrates and fish, which include strong selection pressures, are able to clearly show the adaptive value of maternal effects [125] and behaviours, like aggression [101].

### 4.4. Future studies

Our analyses illustrate a need to diversify species studied to better understand the role life-history traits play in providing context for the potentially adaptive role maternal effects play. Although we found no evidence of publication bias, this effect has only been studied in 17 species across two decades. Primarily, we identified a lack of hormone-mediated maternal effect research with precocial and non-passerine species. There were also several life-history traits we could not analyse due to sample size limitations, such as trophic level or breeding seasonality, as there are too few studies performed with herbivorous or tropical, year-round territorial species. It would also be interesting to look for indications of divergent evolution of the maternal effect response in populations of species that have different life-history strategies; for example, red-necked grebes (*Podiceps grisegena*) can nest in both territorial and colonial patterns [90].

### 4.5. Conclusion

This meta-analysis supports the hypothesis that competition-induced maternal hormone allocation to egg yolks is influenced by life-history traits, suggesting this maternal effect evolved as an adaptation to competition. Colonial species and those with open nests allocate less yolk testosterone in response to competition than solitary and semi-colonial species or species nesting in cavities. Though some of our analyses should be interpreted cautiously owing to sample size limitations, diversifying the number of species studied in future work will only help to further elucidate patterns identified here. Nevertheless, synthetic studies such as ours can help clarify the adaptive role maternally derived hormones play and how they mediate life-history trade-offs. Approaching maternal effects from a life-history perspective will help us understand how variation in this response evolved and elucidate the underlying mechanisms of hormone-mediated maternal effects, an ongoing area of study [126].

## Supplementary Material

Electronic supplementary material: Details of species included in the meta-analysis (Table S1), flow diagram of the inclusion process for published articles in the analysis (Fig. S1), a funnel plot testing for publishing bias (Fig. S2), and all associated references.

## Data accessibility

All data used for analyses are available in the electronic supplementary material, table S1.
