# Supplementary material for: Evolutionary implications of interspecific variation in a maternal effect: a meta-analysis of yolk testosterone response to competition
Source: R Soc Open Sci. 2016 Nov 30;3(11):160499. doi: 10.1098/rsos.160499 (PMC5180134; doi:10.1098/rsos.160499)
Supplement: Electronic supplementary material: Details of species included in the meta-analysis (Table S1), flow diagram of the inclusion process for published articles in the analysis (Fig. S1), a funnel plot testing for publishing bias (Fig. S2), and all associated references. [file rsos160499supp1.pdf]

Supplementary Material for

Evolutionary implications of interspecific variation in a maternal effect: a meta-analysis of yolk testosterone response to competition

Alexandra B. Bentz*, Daniel J. Becker, and Kristen J. Navara

*Corresponding author. E-mail address: abbentz@uga.edu

This PDF file includes:
Table S1
Figs. S1 and S2
References

Table S1. Details of species included in the meta-analysis, including the Fisher's *Z* transformed effect size and both study- and species-specific moderators. Moderators are scored as follows: experiment type, 0 = no manipulation, 1 = indirect manipulation, and 2 = direct, experimental manipulation; coloniality, 1 = solitary, 2 = semi-colonial (1-10 pairs), and 3 = colonial (>10 pairs); nest type, 1 = closed and 2 = open; development type, 1 = altricial and 2 = precocial; and mating type, 1 = monogamous and 2 = polygamous and cooperative breeders. Percent of extra-pair copulations (% EPC) is a weighted average across studies. References for life-history traits follow each species' scientific name. References for specific studies follow corresponding effect size, sample size, and experiment type (if multiple studies within a species used different experiment types). References used to calculate % EPC follow % EPC values.

Scientific name	Effect size (Fisher's Z)	*n*	Experi-ment type	Colon-iality	Nest type	Days to fledge	Clutch size	Dev. type	Mating type	% EPC
Ficedula albicollis[1]	0.53[2]	23[2]	2	1	1	16	6	1	2	39.5[3,4]
Fulica americana[5]	1.07[6]	24[6]	0	1	2	1	7.5	2	1	-
Crotophaga ani[7]	0.16[8]	48[8]	0	2	2	10	5	1	2	-
Tachycineta bicolor[9]	0.71[10], 0.93[10], 0.63[11]	18[10], 10[10], 16[11]	1[10],0[10,11]	1	1	20	5.5	1	1	69.2[12–14]
Athene cunicularia[15]	0.14[16]	47[16]	0	2	1	48.5	8	1	1	-
Passer domesticus[17]	1.00 [18], 0.85[19]	10[18], 23[19]	1[18],2[19]	2	1	14	4.5	1	2	28.7[20,21]
Larus fuscus[22]	-0.06[23]	22[23]	1	3	2	2	3	2	1	-
Guira guira[24]	0.07[25]	35[25]	0	2	2	5.5	6	1	2	-
Parus major[26]	0.13[27]	128[27]	0	1	1	19	8	1	1	29.5[28,29]
Chroicocephalus ridibundus[22]	-0.68[30], -0.76[30]	13[30], 16[30]	0	3	2	10	2.5	2	1	33.3[31]
Hirundo rustica[32]	-0.63[33]	23[33]	0	3	2	20	4.5	1	1	34.7[34]
Sialia sialis[35]	0.73[36], 0.52[37]	28[36], 19[37]	2[36],0[37]	1	1	18	4.5	1	1	25.9[38–40]
Philetairus socius[41]	0.15[42], 0.01[43], 0.05[44]	28[42], 27[43], 18[44]	0	3	2	15	4	1	2	-
Sturnus vulgaris[45]	0.30[46], 0.56[47]	57[46], 24[47]	1[46], 0[47]	2	1	21	4.5	1	2	30.6[48,49]
Taeniopygia guttata[50]	0.35[Bentz et al. unpubl]	16[Bentz et al. unpubl]	2	3	2	15	5	1	1	5.3[51,52]
Coturnix japonica[53]	0.69[54]	20[54]	2	1	2	19	6.5	2	2	-
Sialia mexicana[55]	0.65[56]	20[56]	1	1	1	21	4.5	1	1	45.6[57–59]

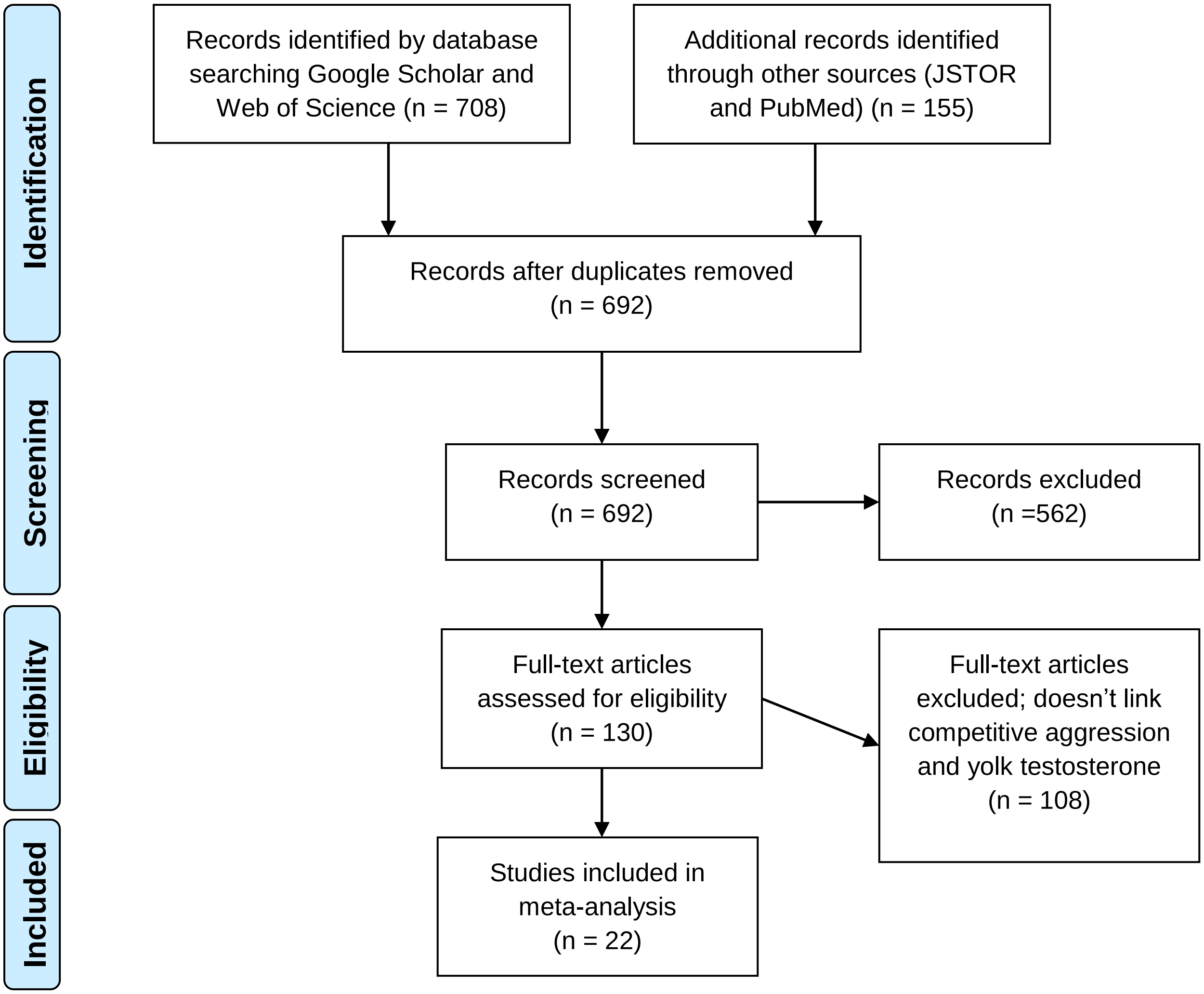

Figure S1. Flow diagram for documenting the data collection and inclusion process according to PRISMA style ([60]; see “Materials and methods” in the main text for detailed criteria for inclusion and exclusion of published articles within our analysis).

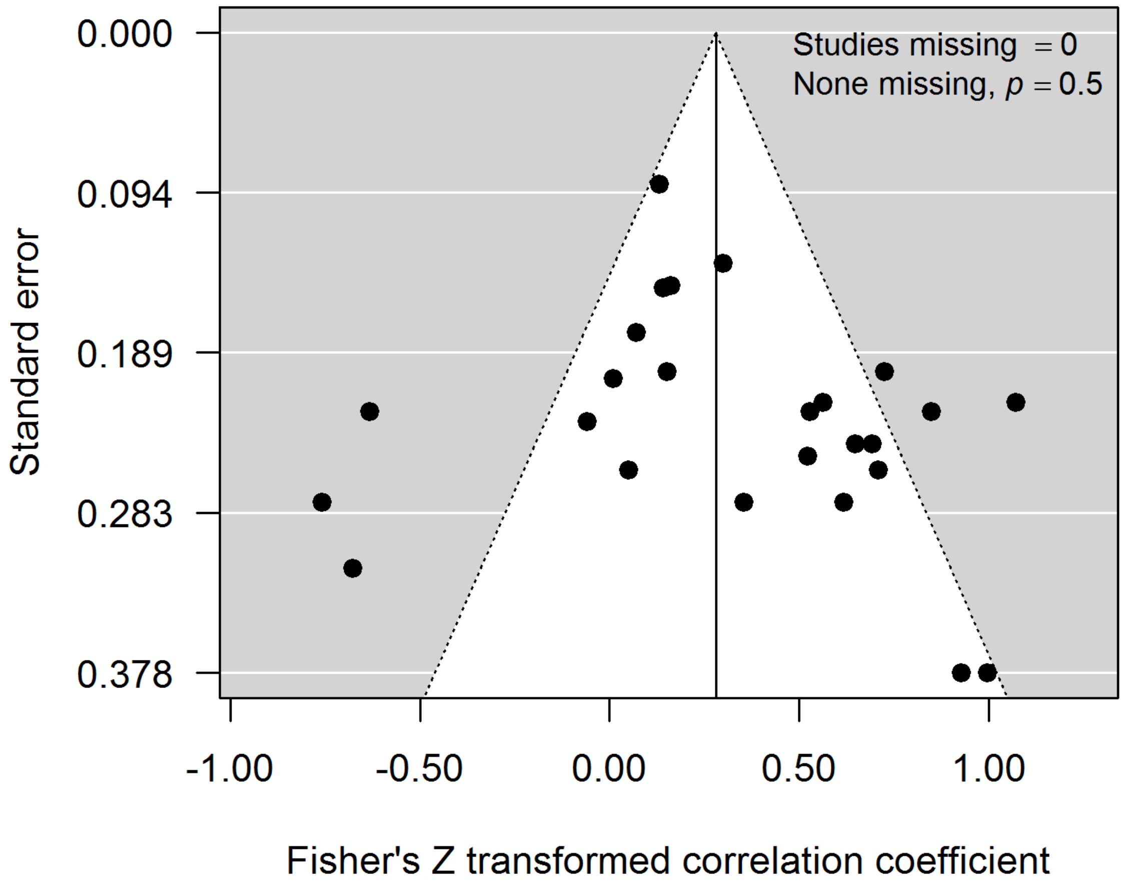

Figure S2. Funnel plot illustrating the relationship between Fisher's *Z* effect size and standard error, where each data point is an individual study on the relationship between competitive environment and yolk testosterone response.

References

1. Cramp S, Perrins CM. 1993 Handbook of the birds of Europe, the Middle East and North Africa. The birds of the Western Palearctic. Vol. VII. Old World Flycatchers to Shrikes. Oxford University Press, Oxford.

2. Hargitai R, Arnold KE, Herényi M, Prechl J, Török J. 2009 Egg composition in relation to social environment and maternal physiological condition in the collared flycatcher. Behav. Ecol. Sociobiol. **63**, 869–882.

3. Rosivall B, Szöllősi E, Hasselquist D, Török J. 2009 Effects of extrapair paternity and sex on nestling growth and condition in the collared flycatcher, *Ficedula albicollis*. Anim. Behav. **77**, 611–617.

4. Sheldon BC, Ellegren H. 1999 Sexual selection resulting from extrapair paternity in collared flycatchers. Anim. Behav. **57**, 285–298.

5. Brisbin IL, Mowbray TB, Pratt HD. 2002 American Coot: *Fulica Americana*. In The Birds of North America (ed A. Poole), Ithaca, NY: The Birds of North America Online.

6. Reed WL, Vleck CM. 2001 Functional significance of variation in egg-yolk androgens in the American coot. Oecologia **128**, 164–171.

7. Quinn JS, Startek-Foote JM. 2000 Smooth-billed Ani (*Crotophaga ani*). In The Birds of North America (eds A. Poole & G. Gill), Philadelphia, PA: The Birds of North America, Inc.

8. Schmaltz G, Quinn JS, Schoech, S J. 2008 Do group size and laying order influence maternal deposition of testosterone in smooth-billed ani eggs? Horm. Behav. **53**, 82–89.

9. Winkler DW, Hallinger K, Ardia D, Robertson R, Stutchbury B, Cohen R. 2011 Tree swallow (*Tachycineta bicolor*). In The Birds of North America (ed A. Poole), Ithaca, NY: The Birds of North America Online.

10. Bentz AB, Navara KJ, Siefferman L. 2013 Phenotypic plasticity in response to breeding density in tree swallows: An adaptive maternal effect? Horm. Behav. **64**, 729–736.

11. Whittingham LA, Schwabl H. 2002 Maternal testosterone in tree swallow eggs varies with female aggression. Anim. Behav. **63**, 63–67.

12. Dunn PO, Robertson RJ, Michaud-Freeman D, Boag PT. 1994 Extra-pair paternity in tree swallows: why do females mate with more than one male? Behav. Ecol. Sociobiol. **35**, 273–281.

13. Kempenaers B, Congdon B, Boag P, Robertson RJ. 1999 Extrapair paternity and egg hatchability in tree swallows: evidence for the genetic compatibility hypothesis? Behav. Ecol. **10**, 304–311.

14. Lifjeld JT, Dunn PO, Robertson RJ, Boag PT. 1993 Extra-pair paternity in monogamous tree swallows. Anim. Behav. **45**, 213–229.

15. Poulin R, Todd LD, Haug EA, Millsap BA, Martell MS, Poole A. 2011 Burrowing owl (*Athene cunicularia*). In The Birds of North America (ed A. Poole), Ithaca, NY: The Birds of North America Online.

16. Welty JL, Belthoff JR, Egbert J, Schwabl H. 2012 Relationships between yolk androgens and nest density, laying date, and laying order in Western Burrowing Owls (*Athene cunicularia hypugaea*). Can. J. Zool. **90**, 182–192.

17. Lowther P, Cink C. 2006 House sparrow (*Passer domesticus*). In The Birds of North America (ed A. Poole), Ithaca, NY: The Birds of North America Online.

18. Schwabl H. 1997 The contents of maternal testosterone in house sparrow *Passer domesticus* eggs vary with breeding conditions. Naturwissenschaften **84**, 406–408.

19. Mazuc J, Bonneaud C, Chastel O, Sorci G. 2003 Social environment affects female and egg testosterone levels in the house sparrow (*Passer domesticus*). Ecol. Lett. **6**, 1084–1090.

20. Stewart IR, Hanschu RD, Burke T, Westneat DF. 2006 Tests of ecological, phenotypic, and genetic correlates of extra-pair paternity in the house sparrow. The Condor **108**, 399–413.

21. Whitekiller RR, Westneat DF, Schwagmeyer PL, Mock DW. 2000 Badge size and extra-pair fertilizations in the house sparrow. The condor **102**, 342–348.

22. Cramp S, Simmons KEL. 1983 Handbook of the birds of Europe, the Middle East and North Africa: the birds of the Western Palearctic. Vol. 3: waders to gulls. Oxford University Press. Oxford, UK.

23. Verboven N, Evans NP, D'Alba L, Nager RG, Blount JD, Surai PF, Monaghan P. 2005 Intra-specific interactions influence egg composition in the lesser black-backed gull (*Larus fuscus*). Behav. Ecol. Sociobiol. **57**, 357–365.

24. Macedo RH. 1992 Reproductive patterns and social organization of the communal Guira Cuckoo (*Guira guira*) in central Brazil. The Auk , 786–799.

25. Cariello MO, Macedo RH, Schwabl HG. 2006 Maternal androgens in eggs of communally breeding guira cuckoos (*Guira guira*). Horm. Behav. **49**, 654–662.

26. Cramp S, Perrins CM, Brooks DJ. 1994 Handbook of the birds of Europe, the Middle East and North Africa: the birds of the Western Palearctic. Vol. 8: crows to finches. Oxford University Press. Oxford, UK.

27. Remeš V. 2011 Yolk androgens in great tit eggs are related to male attractiveness, breeding density and territory quality. Behav. Ecol. Sociobiol. **65**, 1257–1266.

28. Blakey JK. 1994 Genetic evidence for extra-pair fertilizations in a monogamous passerine, the great tit *Parus major*. Ibis **136**, 457–462.

29. Lubjuhn T, Strohbach S, Brun J, Gerken T, Epplen JT. 1999 Extra-pair paternity in great tits (*Parus major*)-a long term study. Behaviour **136**, 1157–1172.

30. Groothuis TG, Schwabl H. 2002 Determinants of within-and among-clutch variation in levels of maternal hormones in black-headed gull eggs. Funct. Ecol. **16**, 281–289.

31. Ležalová-Piálková R. 2011 Molecular evidence for extra-pair paternity and intraspecific brood parasitism in the Black-headed Gull. J. Ornithol. **152**, 291–295.

32. Brown C, Brown M. 1999 Barn swallow (*Hirundo rustica*). In The Birds of North America (eds A. Poole & F. Gill), Ithaca, NY: The Birds of North America Online.

33. Safran RJ, Pilz KM, McGraw KJ, Correa SM, Schwabl H. 2008 Are yolk androgens and carotenoids in barn swallow eggs related to parental quality? Behav. Ecol. Sociobiol. **62**, 427–438.

34. Møller AP, Tegelström H. 1997 Extra-pair paternity and tail ornamentation in the barn swallow *Hirundo rustica*. Behav. Ecol. Sociobiol. **41**, 353–360.

35. Gowaty PA, Plissner JH. 1998 Eastern bluebird (*Sialia sialis*). In The Birds of North America (ed A. Poole), Ithaca, NY: The Birds of North America Online.

36. Navara KJ, Siefferman LM, Hill GE, Mendonca MT. 2006 Yolk androgens vary inversely to maternal androgens in eastern bluebirds: an experimental study. Funct. Ecol. **20**, 449–456.

37. Bentz AB, Sirman AE, Wada H, Navara KJ, Hood WR. 2016. Relationship between maternal environment and DNA methylation patterns of estrogen receptor alpha in wild Eastern Bluebird (*Sialia sialis*) nestlings: a pilot study. Ecol. Evol. **6**, 4741-4752.

38. Gowaty PA, Karlin AA. 1984 Multiple maternity and paternity in single broods of apparently monogamous eastern bluebirds (*Sialia sialis*). Behav. Ecol. Sociobiol. **15**, 91–95.

39. Meek SB, Robertson RJ, Boag PT. 1994 Extrapair paternity and intraspecific brood parasitism in eastern bluebirds revealed by DNA fingerprinting. The Auk , 739–744.

40. Stewart SL, Westneat DF, Ritchison G. 2010 Extra-pair paternity in eastern bluebirds: effects of manipulated density and natural patterns of breeding synchrony. Behav. Ecol. Sociobiol. **64**, 463–473.

41. Sinclair I, Ryan P, Christy P, Hockey P. 2003 Birds of Africa: south of the Sahara. Princeton University Press. Princeton, NJ.

42. Paquet M, Covas R, Chastel O, Parenteau C, Doutrelant C. 2013 Maternal effects in relation to helper presence in the cooperatively breeding sociable weaver. PloS One **8**, e59336.

43. van Dijk RE, Eising CM, Merrill RM, Karadas F, Hatchwell B, Spottiswoode CN. 2013 Maternal effects in the highly communal sociable weaver may exacerbate brood reduction and prepare offspring for a competitive social environment. Oecologia **171**, 379–389.

44. Santos SJD. 2016 Effects of group size on maternal allocation in a colonial cooperatively breeding bird, the sociable weaver. Doctoral dissertation.

45. Cabe P. 1993 European starling (*Sturnus vulgaris*). In The Birds of North America (ed A. Poole), Ithaca, NY: The Birds of North America Online.

46. Eising CM, Pavlova D, Groothuis TG, Eens M, Pinxten R. 2008 Maternal yolk androgens in European starlings: affected by social environment or individual traits of the mother? Behaviour **145**, 51–72.

47. Pilz KM, Smith HG. 2004 Egg yolk androgen levels increase with breeding density in the European starling, Sturnus vulgaris. Funct. Ecol. **18**, 58–66.

48. Pinxten R, Hanotte O, Eens M, Verheyen RF, Dhondt AA, Burke T. 1993 Extra-pair paternity and intraspecific brood parasitism in the European starling, *Sturnus vulgaris*: evidence from DNA fingerprinting. Anim. Behav. **45**, 795–809.

49. Smith HG, von Schantz T. 1993 Extra-pair paternity in the European starling: the effect of polygyny. Condor , 1006–1015.

50. Higgens PJ, Peter JM, Cowling SJ. 2006 Handbook of Australian, New Zealand and Antarctic birds. Vol. 7: boatbill to starlings. Oxford University Press. Oxford, UK.

51. Birkhead TR, Burke T, Zann R, Hunter FM, Krupa AP. 1990 Extra-pair paternity and intraspecific brood parasitism in wild zebra finches *Taeniopygia guttata*, revealed by DNA fingerprinting. Behav. Ecol. Sociobiol. **27**, 315–324.

52. Griffith SC, Holleley CE, Mariette MM, Pryke SR, Svedin N. 2010 Low level of extrapair parentage in wild zebra finches. Anim. Behav. **79**, 261–264.

53. Brazil MA. 1991 Birds of Japan. Smithsonian Institute Press.

54. Guibert F, Richard-Yris M-A, Lumineau S, Kotrschal K, Guémené D, Bertin A, Möstl E, Houdelier C. 2010 Social instability in laying quail: consequences on yolk steroids and offspring's phenotype. PLoS One **5**, e14069.

55. Guinan J, Gowaty P, Eltzroth E. 2008 Western bluebird (*Sialia mexicana*). In The Birds of North America (ed A. Poole), Ithaca, NY: The Birds of North America Online.

56. Duckworth RA, Belloni V, Anderson SR. 2015 Cycles of species replacement emerge from locally induced maternal effects on offspring behavior in a passerine bird. Science **347**, 875–877.

57. Dickinson JL. 2001 Extrapair copulations in western bluebirds (*Sialia mexicana*): female receptivity favors older males. Behav. Ecol. Sociobiol. **50**, 423–429.

58. Dickinson JL. 2003 Male share of provisioning is not influenced by actual or apparent loss of paternity in western bluebirds. Behav. Ecol. **14**, 360–366.

59. Dickinson JL, Akre JJ. 1998 Extrapair paternity, inclusive fitness, and within-group benefits of helping in western bluebirds. Mol. Ecol. **7**, 95–105.

60. Moher D, Liberati A, Tetzlaff J, Altman DG. 2009 Preferred reporting items for systematic reviews and meta-analyses: the PRISMA statement. Ann. Intern. Med. **151**, 264–269.
